# Biogeographical patterns in the structural blue of male *Polyommatus icarus* butterflies

**DOI:** 10.1038/s41598-019-38827-w

**Published:** 2019-02-20

**Authors:** Krisztián Kertész, Gábor Piszter, Zsolt Bálint, László P. Biró

**Affiliations:** 1 Institute of Technical Physics and Materials Science, Centre for Energy Research, P.O. Box 49, H-1525 Budapest, Hungary; 2 Hungarian Natural History Museum, Baross utca 13, H-1088 Budapest, Hungary

## Abstract

Color is a widely used communication channel in the living world for a variety of functions ranging from sexual communication to warning colors. A particularly rich spectrum of colors appears on the wings of many butterflies. The males of lycaenid butterflies often exhibit a conspicuous blue coloration generated by photonic nanoarchitectures on their dorsal wing surfaces. Using UV-VIS spectroscopy, we investigated the spatio-temporal variations of this coloration for *Polyommatus icarus* butterflies, considering an interval of more than 100 years and a geographical range spanning Europe (west) and Asia (east). The blue coloration in Hungary is very stable both within a year (three broods typical in Hungary) and within the period of 100 years (more than 300 generations). East-west geographic variation was investigated among 314 male *P. icarus* butterflies. In agreement with earlier genetic and morphometric studies, it was found that the western males are not divided in distinct lineages. Clear differences in coloration were found between the eastern and western groups, with a transition in the region of Turkey. These differences are tentatively attributed to bottleneck effects during past glaciations.

## Introduction

Structural colors in nature arise from complex interactions between light and the often intricate nanoarchitectures created and optimized over many millennia of biological evolution^1,2^. The diversity of organisms exhibiting structural colors includes butterflies and moths^3^, beetles^4^ and other insects^5^, fishes^6^, birds^7^, mammals^8^ and plants^9^. Because color patterns are a means of communication (e.g., as in mimicry, aposematism, camouflage, and sexual signaling) with other animals, they are among the most visually obvious evolutionary adaptations^10^. In the past decade, the color patterns of insects have emerged as a model system for the study of the interplay between development and evolution^10–12^.

It may be useful to make a distinction between structural and pigment colors. Structural colors are generated by photonic crystal-type^13^ nanoarchitectures^2,14^; these are more broadly defined than photonic crystals, which include only those structures in which a rigorous spatial ordering of the constituent elements is found. In these nanoarchitectures, the existence and spectral position of the photonic ban gap (PBG) depends on the spatial periodicity and the refractive index contrast between the two media, with different refractive indexes building the nanocomposite^2^. In contrast, the absorption of pigments and dyes^15^ is governed primarily by their molecular structure, which is a fixed property of a given substance. Therefore, changing the absorption properties typically involves changing the molecular structure or the chemical properties of the medium–for example, the pH^16^–in which the dye or pigment is contained. However, in living organisms, large changes in pH or other chemical characteristics are generally less likely. The spectral width of the absorption varies from selectively absorbing pigments, such as ommochromes^17^, to broadband absorbers, such as melanin^18^. Selective absorption is responsible for generating a specific color, while broadband absorption in the case of structural colors saturates the color by reducing the amount of stray light or causes the coloration to become dark brown or black (melanization)^19^. In butterflies, pigments are synthesized in the sequence white, red, yellow and black/dark brown^10^. The duration of the synthesis process is typically 1–2 days, and, with a few exceptions, a given scale synthesizes only one pigment^10^.

Butterflies are day-flying insects whose partner-finding strategy is mainly based on visual clues; in butterflies, females have apparently lost the typical sex pheromone glands found in female moths^20^. A blue coloration of structural origin is frequently used by male butterflies for sexual communication, as in several *Morpho*^21^ and *Polyommatus*^22^ species. Recently, it was shown that the spectral position of the blue reflectance peak of *Polyommatus icarus* males within a certain population shows unexpectedly low variation (of the order of only 10 nm)^23^. In addition, this blue sexual communication color exhibits substantially lower variation in response to strong environmental stresses, such as prolonged cold shock, than do pigment-based patterns on the ventral wing surfaces of *P. icarus* butterflies^24^.

We aimed to determine the variation of structural and pigment-based colors of *P. icarus* over a large geographical range, as climatic and biogeographical conditions vary extensively over the distribution range of this butterfly species, which covers almost all of the Palearctic region. We used the butterfly collection of the Hungarian Natural History Museum (HNHM) as source of samples. Before examining geographic variation, we investigated the structural color differences among the three broods that develop within a year and the stability of the blue coloration of males over a period exceeding 100 years. The spectroscopic method we developed previously and applied here for the precise measurement of structural coloration is in no way destructive to museum exemplars and is suitable for measuring large numbers of individuals from museum collections^25,26^.

Variation in the pigment-based pattern on the ventral wing surface of *P. icarus* butterflies has been investigated previously^27^; therefore, we restricted our investigation to the blue generated by photonic nanoarchitectures in the males. Artemyeva^27^ quantified the overall phenodeviation of the ventral pigment-based pattern of *P. icarus* butterflies and found that European exemplars exhibited a lower frequency of phenodeviation than did Asian ones. In an earlier work, she investigated the geographic variations of individual elements of the pigment-based pattern on the ventral side of the wings (e.g., bright white stroke in cells M2–M3–Cu1 of the lower side of the posterior wing, reduced and aligned row of postdiscal ocelli on the lower side of the posterior wing). She found that two large groups of *P. icarus* populations could be distinguished based on 16 characters: a western group and an eastern group^28^. Our present results, based on the spectral position of the blue sexual signaling color of structural origin, are in agreement with these findings and indicate that the eastern and western populations possess distinct characteristics, which can be measured precisely using spectroscopic methods.

## Results

**Figure 1:**
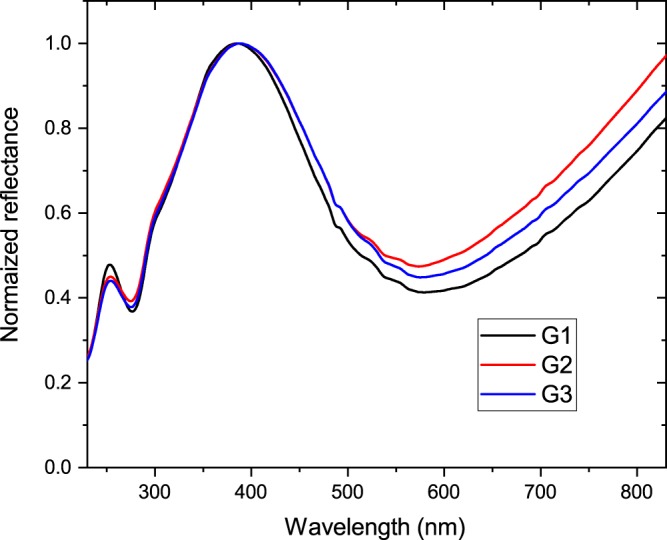
Normalized reflectance measured on the dorsal wing surfaces of male *Polyommatus icarus* butterflies from the three broods of the year 2014. G1: first brood, G2: second brood, G3: third brood.

The butterfly species *P. icarus* has three broods per year over a large fraction of Europe. We used samples from region Sălaj (Romania), to examine the differences in male structural color among the generations flying in April-May, July-August and September-October. The spectra of 10 individuals were averaged from each brood from the year 2014, and the spectra were normalized to the blue reflectance maximum to facilitate comparison. As shown in Fig. 1, the three spectra overlap almost completely in the spectral range from 300 to 600 nm.

**Figure 2:**
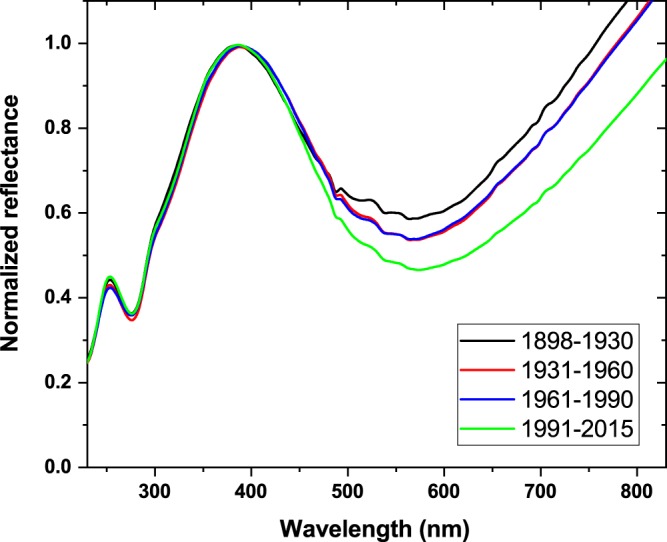
Normalized reflectance measured on the dorsal wing surface of male *Polyommatus icarus* butterflies from a period of 117 years. The 24-25 data points from each of the periods indicated in the legend were averaged to facilitate comparison.

Next, we examined the temporal stability of the structural color over a period greater than 100 years, equivalent to more than 300 generations. The sampling was restricted geographically to the environs of Budapest (cca. 30 km radius) and to the summer (2nd) generation. The period from 1898 to 2015 was divided into four sub-periods such that each sub-period included 24–25 individuals. As shown in Fig. 2, the spectra normalized to the blue reflectance maximum coincide very well. A gradual reduction in melanin content (spectral range 500 to 800 nm) from the newest to the oldest samples was observed, which we attribute to slow melanin decomposition over time.

**Figure 3:**
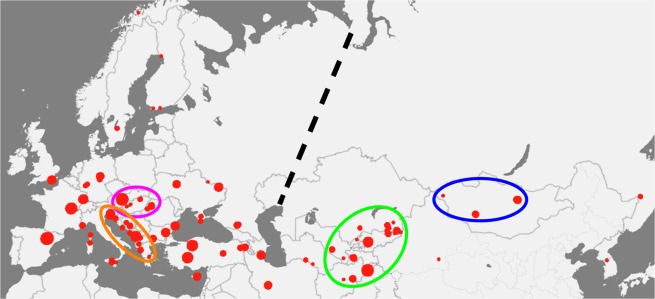
The geographical source locations of the samples analyzed are indicated by red dots. The size of a dot is proportional to the number of the samples from that location. Four test regions were selected and are encircled on the map: two from Europe (Central East European Plane (magenta), Adriatic Coast (orange)) and two from Asia (Mongolian Steppe (blue) and Central Asia (green)). The broken black line approximately marks the location of the Ural Mountains, separating Europe and Asia.

**Figure 4:**
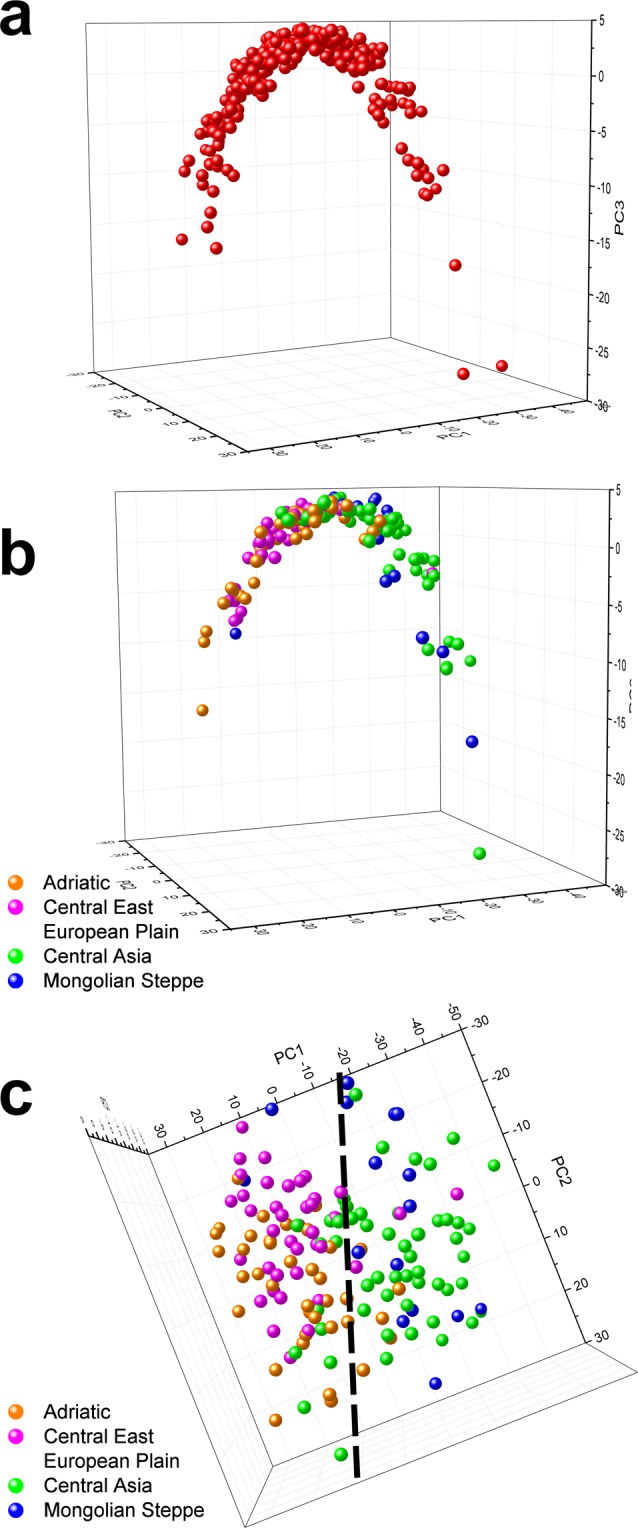
PCA results of the averaged spectra (8 measurements, 2 per wing) of the dorsal wing surfaces of male *Polyommatus icarus* butterflies from Europe and Asia. Each individual is represented by the averaged spectrum and the corresponding dot in the 3D space defined by the first three PCs. (**a**) The PCA score plot for all 314 specimens; the dots follow a parabolic-like shape. (**b**) The PCA scores of the averaged spectra of individuals from the four selected test regions marked in Fig. 3: Central East European Plain (magenta), Adriatic Coast (orange), Mongolian Steppe (blue) and Central Asia (green). (**c**) Top view of the distribution. An apparent separation line between Europe and Asia is marked by a black dashed line. (See Supplementary Movie for (**b**,**c**) evolution).

**Figure 5:**
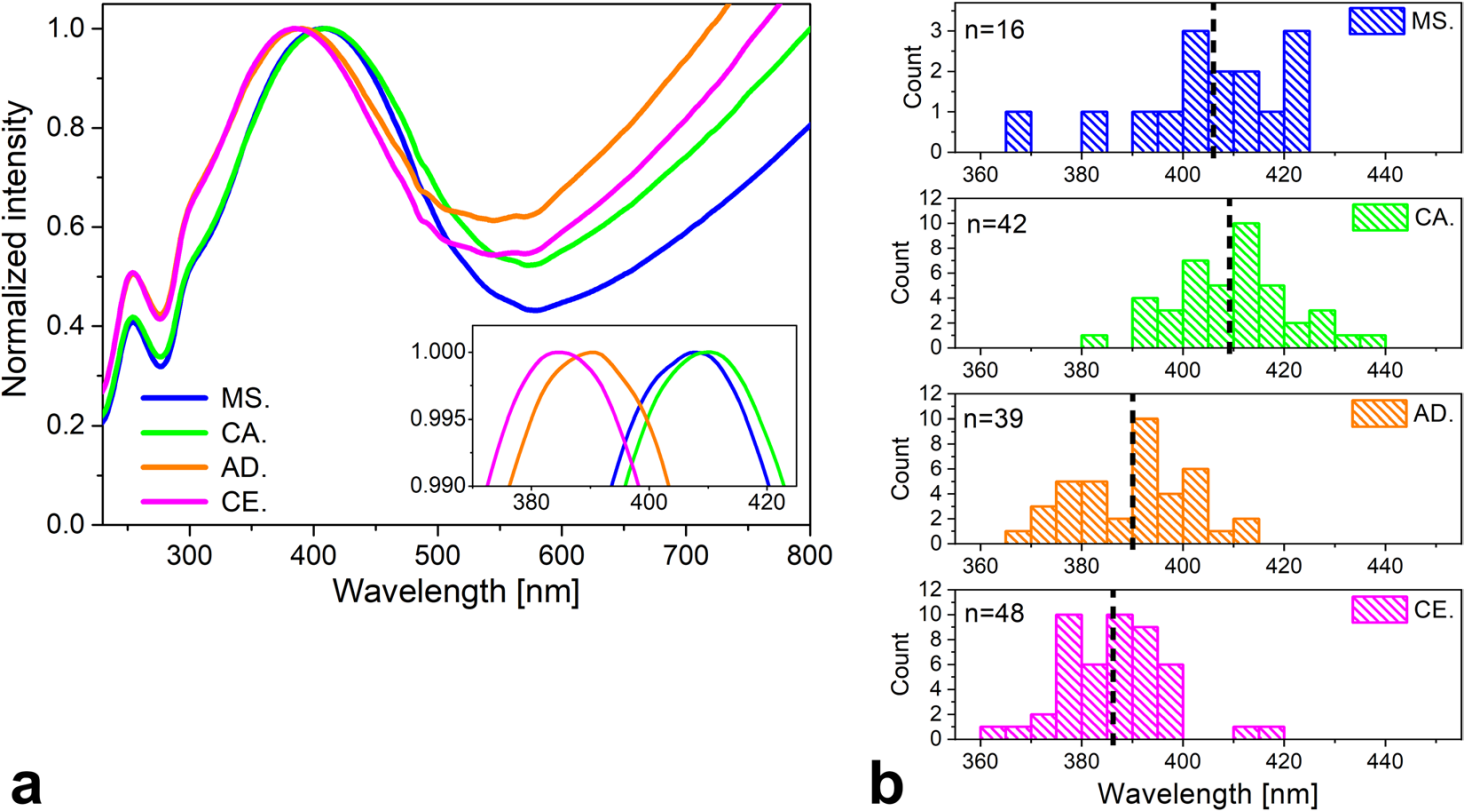
Spectra of *Polyommatus icarus* butterflies from the four regions marked in Fig. 3. (**a**) Spectra averaged over all of the individuals and all of the measurements for a given region. Strong overlap in the spectral position of the blue reflectance maximum of the European (magenta and orange) and Asian (blue and green) samples is apparent; inset: magnified view of the blue peak region. (**b**) Histograms of the spectral position of the blue reflectance maximum of the samples from the four regions; the difference in the spectral position of the blue reflectance maximum in (**a**) is fully supported by the histograms, which show the spectral position of the averaged spectra of the individual samples. The numerical value in the left upper corner of each histogram indicates the number of individual samples for that histogram.

To investigate the biogeographic differences, we selected 314 samples from the collection of the HNHM. As shown in Fig. 3, the range covered by these samples extends to Scandinavia in the north, England in the west, Sicily in the south and South Korea in the east. For each specimen, two measurements were taken on each wing, and the 8 individual spectra were averaged. After normalization to the blue reflectance maximum, the resulting averaged spectrum was analyzed by principal component analysis (PCA)^29^. The 3D graph produced by plotting the 314 samples according to their first three principal components (PCs) is shown in Fig. 4a. One can clearly distinguish both sides of the parabolic-like shape. To test for geographic differences, another graph was drawn using only the points corresponding to the four selected test regions marked in Fig. 3. This graph is presented in Fig. 4b. As shown in Fig. 4c, a separation between the European and Asian spectra was found. Two more operations were performed with the spectra from the four test regions: all of the spectra from a given test region were averaged and compared with the averaged spectra of the other test regions (Fig. 5a), and the spectral position of the maximum of the blue reflectance peak for the individual specimens in each test region was used to construct the histograms in Fig. 5b.

As shown in Fig. 5, if averaging the spectra in each of the four test regions, the averaged spectra in Europe and in Asian are very similar, and the spectral positions of the reflectance maxima differ by about 20 nm between Europe and Asia.

## Discussion

Color polymorphisms in natural populations present an ideal opportunity to study the evolutionary drivers of phenotypic diversity. The homogenous blue coloration of the dorsal wing side of *Polyommatus icarus* males can be precisely measured using appropriate spectrometers. This possibility, together with the wide range of this butterfly species, which extends over the majority of the Palearctic region, makes this butterfly a valuable test species to demonstrate the potential of the proposed method. Our method of carrying out precise spectral measurements on set museum exemplars without damaging them^25,26^–as achieved here–can be used to rigorously and quantitatively investigate both the spatial and temporal variations in the coloration of various butterfly species using large numbers of samples. A further advantage of the method is that the spectral data collected can be conveniently evaluated by well-established mathematical methods such as PCA.

The phenotypic diversity of the butterfly species *P. icarus* was investigated by Artemyeva^27,28^ based on the pigment-based pattern characteristics of the ventral wing surfaces. Statistically significant differences were found between the European (western) and Asian (eastern) exemplars. However, the precise quantification of such complex patterns may be challenging. Spectral methods can be very useful as they allow precise measurement of the reflectance spectrum using a fiber optic spectrometer^23^ and a high level of measurement reproducibility. Moreover, the fiber optics allow the measurements to be performed only over certain regions of the butterfly wing (down to a few mm^2^) if desired, making it possible to investigate wings with non-homogenous coloration.

Within a geographical region, the temporal data clearly show that the structural blue of *P. icarus* is very stable, both over the three broods within a year and over a period exceeding 100 years. This result is in good agreement with our earlier finding that outlier colors are infrequent^23^. In addition, strong environmental stress (subjecting of pupae to 5 °C for more than 60 days) that greatly modified the pigment-based pattern on the ventral wing surface of *P. icarus* males induced only minor modifications in the structural blue^24^.

To examine the patterns among biogeographical regions in greater detail, we selected several regions. In Europe, we selected two regions: the Adriatic region (circled in orange in Fig. 3), which comprises, according to the classification of biogeography provinces of Udvardy^30,31^, Mediterranean Sclerophyll and Balkan Highlands; and the Central East European Plain (circled in magenta in Fig. 3), comprising the Middle European Forest and the Pannonian Plain. In Asia, we selected the Central Asian region (circled in green in Fig. 3), comprising parts of the Turanian region, Anatolian-Iranian-Desert, and Hindu Kush Highlands. The fourth region is the Mongolian Steppe (circled in blue in Fig. 3), including parts of the Mongolian-Manchurian Steppe, Takla-Makan-Gobi Desert and Pamir-Tien-Shan Highlands.

In Fig. 4, the PCA results of the averaged spectra (8 measurements, 2 per wing) of the male *P. icarus* butterflies from Europe and Asia are shown. Each averaged spectrum is represented by one dot in the 3D space defined by the first three PCs. The PCA scores (dots) for the 314 specimens form a parabolic-like shape (Fig. 4a). To obtain more insight regarding the geographic distribution, the dots for the averaged spectra of the individuals from the four selected test regions marked in Fig. 3 were plotted: Central East European Plain (magenta), Adriatic Coast (orange), Mongolian Steppe (blue) and Central Asia (green) (Fig. 4b). When viewing this “saddle” from above, the separation between Europe and Asia becomes apparent–marked by a black broken line in Fig. 4c.

When comparing the averaged spectra of the samples originating from the four selected test regions (Fig. 5a), one can observe that the spectra for the selected European regions overlap with each other, as do the spectra for the selected regions from Asia. Clear differences are present between the European and the Asian spectra. Moreover, examination of the histograms in Fig. 5b reveals that the shift in peak position between the eastern and western individuals is not an effect of averaging, as the distributions also have different center points.

According to the Köppen-Geiger climatic classification^32^, the Adriatic region has a humid subtropical and Mediterranean climate (Csa, Cwa), the Central East European Plain has a humid continental climate (Dfb), the Central Asian region has a tropical and subtropical desert climate (mainly BWk and Csa with some BSk), and the Mongolian Steppe is in mid-latitude steppe and has a desert climate (mainly BSk with some BWk). Despite the differences in climatic conditions among regions, the spectra corresponding to the European butterflies and Asian butterflies overlap within the continent and exhibit clear differences between Asia and Europe.

The temporal investigation presented here and the previous stress experiments clearly demonstrated the stability of the blue sexual signaling color. However, the similarities and dissimilarities discussed above cannot be unambiguously associated with biogeographical regions or climatic features. The likely explanation for the measured spectral differences in the blue color between the western and eastern *P. icarus* males may be found in the history of the species during the glaciations of the past 2 million years^33^. It is likely that during past glacial periods, the eastern *P. icarus* butterflies, in a similar manner as their western counterparts, were restricted to a few refuges from which they subsequently and repeatedly recolonized warming regions. Genetic drift in the bottlenecked populations^34^ may have induced the differences in the blue color between the eastern and western *P. icarus* males and been followed by the fixation of the differences by non-random mating, as the blue color is a sexual signaling color. During these repeated withdrawal and recolonization waves, the limited gene flow between east and west due to geographic barriers, caused the evolution of two slightly different genetic pools. These different pools are equally manifested in the differences in the blue sexual signaling color and in the elements of the pigment-based pattern on the ventral wing surfaces of *P. icarus* butterflies^27,28^.

**Figure 6:**
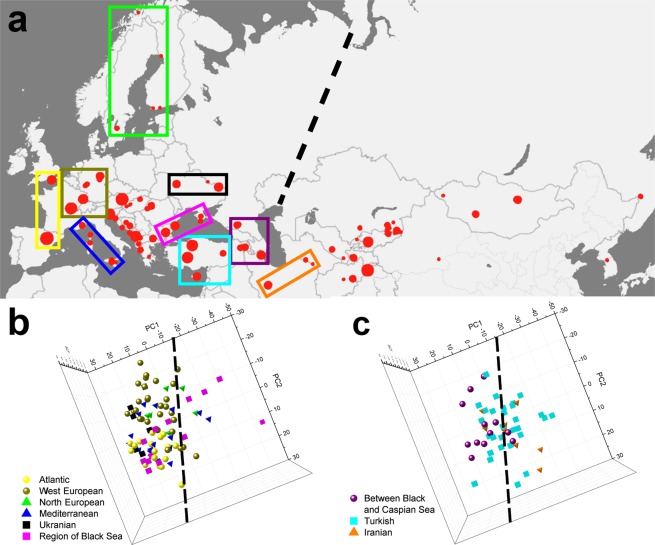
(**a**) Location of the sample categories for geographical regions not presented in Fig. 3. (**b**) The corresponding positions of the individual specimens from these regions in the PCA scores plot.

To further examine the spectral differences between western and eastern populations of *P. icarus*, we examined by PCA a few more regions outlined in Fig. 6: the Atlantic region (yellow rectangle), the North European region (light green rectangle), the West European region (dark green rectangle), the Mediterranean region (dark blue rectangle), the region around the Black Sea (magenta rectangle), the Ukrainian region (black rectangle), the Turkish region (light blue rectangle), the region between the Black and Caspian Seas (violet rectangle) and the Iranian region (orange rectangle). To avoid cluttering the figure, only the line separating the eastern and western regions is included from Fig. 4c.

The Atlantic and North European samples are found on the western side of the East-West Separation Line (EWSL). Most of the Mediterranean samples fall also on the western side of the EWSL, but a fraction is found on the eastern side of the line. This finding is also apparent in the Turkish samples, which are approximately equally distributed between the two sides of the EWSL. The samples originating from the region between the Black and Caspian seas exhibit a pronounced western character, while the Ukrainian samples are all found on the western side of the EWSL. The Iranian samples are placed predominantly on the eastern side of the EWSL. These results indicate that gene flow between the eastern and the western groups is mainly occurring in the region of the Black Sea and Turkey.

The above findings are in good agreement with results on the phenotypic variation of the ventral wing patterns. When investigating *P. icarus*, Artemyeva found, as summarized in Fig. 1 of that paper^27^, that the phenotypic diversity of the wing pattern was higher in populations along the southeastern boundary of the range than in those from northwestern and central regions. In a more detailed investigation^28^ of selected elements of the overall pattern on the ventral wings, two large groups of *P. icarus* populations, western and eastern, were distinguished. The separation line between these two groups as one can infer from the maps published by Artemyeva and the separation line found by PCA in the present study coincide with each other and with the chain of geographic barriers separating Asia from Europe, i.e., the Black Sea, Caucasus Mountains, the Caspian Sea and the Ural Mountains. According to our data, the territory of Turkey acts as a channel, allowing gene flow between the two continents.

A similar distinction of western and eastern groups within Europe is found in another lycaenid butterfly: *Polyommatus coridon*. Detailed genetic investigations revealed that as a consequence of postglacial range expansion, two distinct lineages are found, a western lineage and an eastern lineage, separated by a contact zone that spans from the sandy areas of north-eastern Germany to along the mountain ranges of the German–Czech border and throughout the eastern Alps. The mountain ranges between Bavaria and Bohemia represented a strong barrier to hybridization of the two lineages in this region. Higher levels of hybridization were found in the populations of the eastern Alps, especially in the northeastern region, where the Danube may have acted as an expansion corridor for both lineages^34^. A recent DNA-based reconstruction of the phylogeny of the butterfly genus *Lysandra* (Lycaenidae, Polyommatinae) also found that the western and eastern lineages constitute two distinct groups^35^.

The patterns we found in the distribution of *P. icarus* groups exhibit similarities with those reported earlier for the impact of glacial-interglacial range-shifts of thermophilic (butterfly) species, and the persistence in distinct glacial refugia (for *Melanargia galathea* and *Maniola jurtina*, (both Satyrinae: Nymphalidae) and *P. icarus*)^36–40^.

An examination of the genetic diversity of *P. icarus* in Europe revealed that unlike *P. coridon*, *P. icarus* shows no clear differentiation into different genetic lineages^41^. Compared to other butterflies and moths, *P. icarus* showed very high genetic diversity at the population level in that study, while genetic differentiation among samples at the continental level was very low. A recent study focusing on the complex biogeographical history of *P. icarus* and *Polyommatus celina* butterflies reported that over all of Europe except the region around the Mediterranean, the genitalia of *P. icarus* are similar and that significant differences begin to appear in eastern Turkey^33^. These reports support our interpretation of the origin of the observed biogeographic differences in the structural blue of *P. icarus* males.

As with many other lycaenid butterflies, *P. icarus* presents strong sexual dimorphism, which is manifested primarily in the color of the dorsal wing surface: structural blue in males and pigment brown in females. The complex pattern on the ventral wing surfaces is almost identical between the females and males, although the background is slightly darker in females. It is unlikely that this pattern plays an important role in sexual communication; rather, it is more likely that it has a role in crypsis, when the butterflies rest with closed wings, as both the males and females are subjected to the same predators.

Previously, we showed that the blue color and underlying nanoarchitecture are species specific in nine closely related polyommatine lycaenid species living in the same habitat^22^. The blue color of the males of sexually dimorphic lycaenids is used in sexual communication, both male to male and male to female. The many cases of sexual dimorphism in butterflies present a strong signature of sexual selection in that the males exhibit the more visually striking color patterns^42^. Our finding that within a given population of *P. icarus*, the spectral position of the blue reflectance maximum does not show a normal (Gaussian) distribution–as would be expected for random variation–but a narrow, uniform distribution with a range of ±10 nm around the mean value^23^ indicates that the blue color is subject to pronounced sexual selection. These sexual signaling colors are sometimes presented to females via highly ritualized aerial courtship routines, which supports the hypothesis that they evolved as mating signals. There is good evidence across many species that such color patterns are used by females to recognize potential mates and assess their genetic quality^42^. Several studies also demonstrate mate discrimination by females based on the expression of the male color signals, which suggests that the functions of these traits extend beyond species and/or sexual identity^43,44^. It is possible that female butterflies obtain indirect benefits from mating with more “properly” colored males, for example, by generating more attractive and/or viable offspring due to receiving “good” or “complementary” genes from their partner^42^.

The difference in the averaged spectral position of the blue color, shown in Fig. 5a, places the eastern and the western males at the two ends of a 20 nm range. This difference, which exceeds the degree of intra-population variation by a factor of 2, is not obviously associated with climatic differences. This is particularly evident when considering that the climatic differences of the North European region (light green rectangle in Fig. 6) from the Mediterranean region (dark blue rectangle in Fig. 6) are much more pronounced than are those of the former region from the East European Plane and the Central Asian region. *P. icarus* has adapted to these differences by adjusting the number of broods per year: two broods in Southern Scandinavia^45^ and up to five broods in the region of the Mediterranean Sea^46^.

It has been shown experimentally in *P. icarus* that the blue structural color is much more stable than the pigment-based pattern on the ventral wing side following exposure to prolonged environmental stress^24^. However, it is possible that during bottleneck periods, females of the eastern and western populations began selecting for different spectral positions of the blue reflectance maximum. As gene flow was limited or absent between the two regions, different color preferences evolved in the east and west. This possibility becomes more likely if we assume that the females have a “preference range”, for example, spanning 10 nm, similar to the range of variation in male blue color.

Concerning the formation of “local colors”, it is worth to point out that this may occur much more easily in the case of physical colors than for pigment colors. In the latter case, the chemical structure of the pigment molecule has to be modified to change the absorption, while for structural colors, even in the simplest model of multilayers, a modification of the layer dimensions by only 10 nm may produce a shift of 10–20 nm in the spectral position of the reflectance maximum. The modification of the chemical structure of a pigment molecule most often necessitates the modification of a complex biochemical synthesis route. The periodicity of the nanoarchitectures generating the structural colors is generated by the self-assembly of constituents inside the cell. The details of the self-assembly process are not yet fully understood. We showed in an earlier work^22^, that the species-specific blue colors of the males of nine closely related lycaenid butterflies are all generated by the same pepper-pot-type nanoarchitecture. It was possible to use the species-specific spectra and an artificial neural network software to identify the species with an accuracy of 96% and on the basis of the structural parameters of the nanoarchitectures we could identify the species with an accuracy of 91%.

In summary, we developed a new, robust method for examining color differences in a butterfly species over wide geographical and temporal ranges. Using *P. icarus* as a test species, we showed that the blue sexual signaling color of structural origin in males exhibited stability both over the course of a year (3 broods tested) and over a period of >100 years. PCA revealed intra-continental similarity in the spectral characteristics of eastern (Asiatic) and western (European) populations, while the eastern and western populations clearly showed differences in the spectral position of the blue reflectance maxima. The observed differences were attributed to population bottlenecks that occurred during past glaciations.

## Methods

### Animals and collections

*Polyommatus icarus* (Rottemburt, 1775) (Polyommatinae: Lycaenidae) males were obtained from the scientifically curated Lepidoptera collection of the Hungarian Natural History Museum. All of the specimens had been stored there under the same conditions in drawers of museum cabinets to prevent photobleaching. Detailed information about the exemplars can be found in the supplementary information (Supplementary Table).

### Reflectance measurements

The wing reflectance of the investigated specimens was measured using an UV-VIS-NIR spectrometer (Avantes AvaSpec-HS1024TEC) with perpendicular white light illumination (AvaLight-DH-S-BAL deuterium halogen source) and detection. The standard Avantes reflection probe (Avantes FCR-7UV200-2-ME-SR), complemented with the device we developed, called a “spectroboard”^26^, allowed us to conduct reflectance measurements without harming the museum exemplars, as the probe was moved above the wings of the pinned butterflies while we recorded the spectral data. The detected light was analyzed over the wavelength range of 200 to 1100 nm. During the measurements, the Avantes white diffuse tile (Avantes WS-2) was used as a reference.

### Statistical analysis

Principal Component Analysis (PCA)^29^ and all the data visualization were carried out using Originlab OriginPro 2018 software.

## Supplementary information

Supplementary video

Supplementary table

## Data Availability

All the data are available upon request.
