# Supplementary figures and images for: Biogeographical patterns in the structural blue of male Polyommatus icarus butterflies
Source: Sci Rep. 2019 Feb 20;9:2338. doi: 10.1038/s41598-019-38827-w (PMC6382816; doi:10.1038/s41598-019-38827-w)

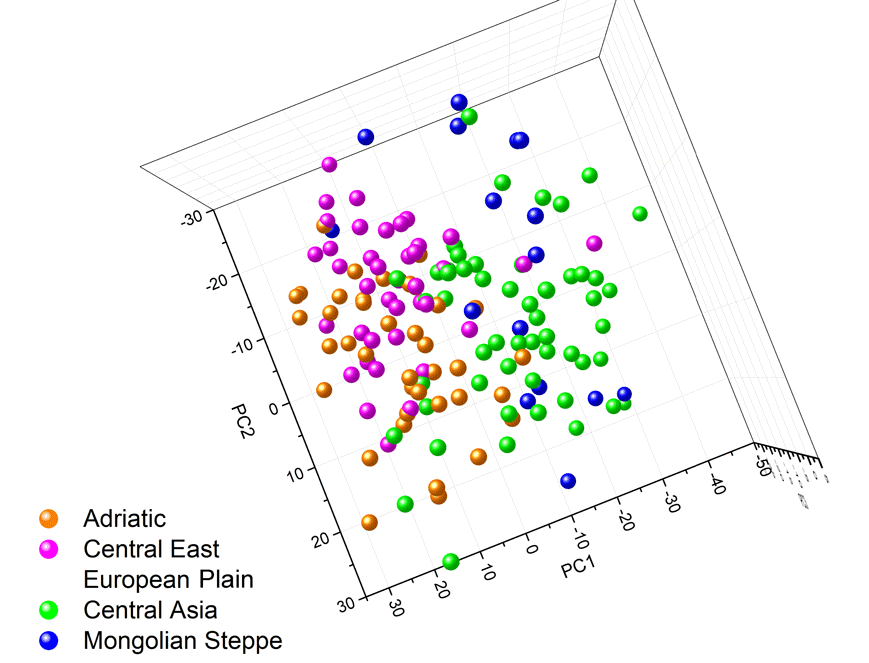

Supplement: Supplementary file 1 — Supplementary video [file 41598_2019_38827_MOESM1_ESM.gif]
