# Supplementary material for: Biogeographical patterns in the structural blue of male Polyommatus icarus butterflies
Source: Sci Rep. 2019 Feb 20;9:2338. doi: 10.1038/s41598-019-38827-w (PMC6382816; doi:10.1038/s41598-019-38827-w)
Supplement: Supplementary file 2 — Supplementary table [file 41598_2019_38827_MOESM2_ESM.docx]

Supplementary table

Detailed information about the Polyommatus icarus butterfly exemplars

Biogeographical patterns in the structural blue of male Polyommatus icarus butterflies

Krisztián Kertész, Gábor Piszter, Zsolt Bálint and László P. Biró

| **Nr.** | **Country** | **City** | **Year** | **Month** | **Day** | **Collector** |
| --- | --- | --- | --- | --- | --- | --- |
| 1 | Romania | Jebucu | 2014 | 5 | 22 | Bálint Zs., Katona G. |
| 2 | Romania | Jebucu | 2014 | 5 | 22 | Bálint Zs., Katona G. |
| 3 | Romania | Jebucu | 2014 | 5 | 22 | Bálint Zs., Katona G. |
| 4 | Romania | Buciumi | 2014 | 5 | 23 | Bálint Zs. |
| 5 | Romania | Buciumi | 2014 | 5 | 23 | Bálint Zs. |
| 6 | Romania | Buciumi | 2014 | 5 | 23 | Bálint Zs. |
| 7 | Romania | Cuzăplac | 2014 | 5 | 23 | Bálint Zs. |
| 8 | Romania | Pria | 2014 | 5 | 22 | Bálint Zs., Katona G. |
| 9 | Romania | Pria | 2014 | 8 | 12 | Katona G. |
| 10 | Romania | Pria | 2014 | 8 | 12 | Katona G. |
| 11 | Romania | Clit | 2014 | 8 | 14 | Katona G. |
| 12 | Romania | Clit | 2014 | 8 | 14 | Katona G. |
| 13 | Romania | Iaz | 2014 | 9 | 30 | Bálint Zs., Katona G. |
| 14 | Romania | Băbiu | 2014 | 10 | 1 | Bálint Zs., Katona G. |
| 15 | Mongolia | Bayan-Olgii | 2005 | 7 | 1 | Benedek B. Csővári T. |
| 16 | Mongolia | Bayangol | 2008 | 6 | 28 | Benedek B. |
| 17 | Mongolia | Bayangol | 2008 | 6 | 28 | Benedek B. |
| 18 | Mongolia | Jargalant | 2008 | 6 | 26 | Benedek B. |
| 19 | Mongolia | Bayangol | 2008 | 6 | 27 | Benedek B. |
| 20 | Mongolia | Bayangol | 2008 | 6 | 28 | Benedek B. |
| 21 | Mongolia | Jargalant | 2008 | 6 | 25 | Benedek B. |
| 22 | Mongolia | Bayangol | 2008 | 6 | 27 | Benedek B. |
| 23 | Mongolia | Jargalant | 2008 | 6 | 25 | Benedek B. |
| 24 | Mongolia | Jargalant | 2008 | 6 | 26 | Benedek B. |
| 25 | Kazahstan | Türgen | 2009 | 7 | 16 | Benedek B. |
| 26 | Kazahstan | Ile Alataūy Zhotasy | 2002 | 9 | 22 | Benedek B. |
| 27 | Kazahstan | Talghar | 1994 | 5 | 23 | Fábián Gy., Retezár I. |
| 28 | Kazahstan | Qapshaghay | 2007 | 6 | 11 | Benedek B. |
| 29 | Kazahstan | Qapshaghay | 2007 | 6 | 11 | Benedek B. |
| 30 | Kazahstan | Qapshaghay | 2007 | 6 | 11 | Benedek B. |
| 31 | Kazahstan | Kegen | 2007 | 6 | 17 | Benedek B. |
| 32 | Kazahstan | Usharal | 2007 | 6 | 12 | Benedek B. |
| 33 | Kazahstan | Narynkol | 2007 | 6 | 16 | Benedek B. |
| 34 | Kazahstan | Kegen | 2006 | 5 | 28 | Benedek B. |
| 35 | Kazahstan | ulitsa Altyn-Emel | 1995 | 8 | 14 | Varga Z., Fábián Gy. |
| 36 | Kazahstan | Usharal | 2007 | 6 | 12 | Benedek B. |
| 37 | Kazahstan | Kegen | 2006 | 5 | 28 | Benedek B. |
| 38 | Kazahstan | Ile Alataūy Zhotasy | 2002 | 9 | 22 | Benedek B. |
| 39 | Kazahstan | Kegen | 2007 | 6 | 17 | Benedek B. |
| 40 | Turkmenistan | Ashgabat | 1986 | 6 | 15 | Dubatolov |
| 41 | Turkmenistan | Dushak | 1988 | 8 | 27 | Zintsenko |
| 42 | Turkmenistan | Ashgabat | 1986 | 6 | 12-13 | Dubatolov |
| 43 | Kyrgyzstan | Ichke-Jergez | 2007 | 7 | 20 | Biophot expedition |
| 44 | Kyrgyzstan | Ysyk-Köl | 2007 | 7 | 21 | Biophot expedition |
| 45 | Kyrgyzstan | Gul'ma | 2007 | 7 | 9 | Biophot expedition |
| 46 | Kyrgyzstan | Gul'ma | 2007 | 7 | 9 | Biophot expedition |
| 47 | Kyrgyzstan | Gul'ma | 2007 | 7 | 9 | Biophot expedition |
| 48 | Kyrgyzstan | Ysyk-Köl | 2007 | 7 | 21 | Biophot expedition |
| 49 | Kyrgyzstan | Gul'ma | 2007 | 7 | 9 | Biophot expedition |
| 50 | Kyrgyzstan | Gul'ma | 2007 | 7 | 9 | Biophot expedition |
| 51 | Kyrgyzstan | Gul'ma | 2007 | 7 | 9 | Biophot expedition |
| 52 | Kyrgyzstan | Gul'ma | 2007 | 7 | 9 | Biophot expedition |
| 53 | Kyrgyzstan | Ysyk-Köl | 2007 | 7 | 21 | Biophot expedition |
| 54 | Kyrgyzstan | Gul'ma | 2007 | 7 | 9 | Biophot expedition |
| 55 | South Korea | Pocheon | 1975 | 7 | 27 | Papp J., Vojnits A. |
| 56 | Russia | Khabarovsk | 1937 | 7 |  | Coll. Velez |
| 57 | Russia | Khabarovsk | 1937 | 7 |  | Coll. Velez |
| 58 | Pakistan | Shandūr Pass | 1994 | 7 | 12 | Herczig B, László M. Gy., Ronkay G. |
| 59 | Pakistan | Shandūr Pass | 1994 | 7 | 12 | Herczig B, László M. Gy., Ronkay G. |
| 60 | Pakistan | Shandūr Pass | 2000 | 6 | 24-25 | Varga Z., Ronkay G. |
| 61 | Pakistan | Shandūr Pass | 2000 | 6 | 26-27 | Varga Z., Ronkay G. |
| 62 | Pakistan | Shandūr Pass | 2000 | 6 | 26-27 | Varga Z., Ronkay G. |
| 63 | Pakistan | Shandūr Pass | 2000 | 6 | 26-27 | Varga Z., Ronkay G. |
| 64 | Pakistan | Shandūr Pass | 1994 | 7 | 12 | Herczig B, László M. Gy., Ronkay G. |
| 65 | Pakistan | Shandūr Pass | 1994 | 7 | 12 | Herczig B, László M. Gy., Ronkay G. |
| 66 | Pakistan | Shandūr Pass | 1994 | 7 | 13 | Herczig B, László M. Gy., Ronkay G. |
| 67 | Pakistan | Shandūr Pass | 1994 | 7 | 13 | Herczig B, László M. Gy., Ronkay G. |
| 68 | Pakistan | Shandūr Pass | 1994 | 7 | 13 | Herczig B, László M. Gy., Ronkay G. |
| 69 | Mongolia | Orog Lake | 1988 | 8 | 3 | Szabóky Cs. |
| 70 | Mongolia | Orog Lake | 1988 | 8 | 3 | Szabóky Cs. |
| 71 | Mongolia | Orog Lake | 1988 | 8 | 3 | Szabóky Cs. |
| 72 | Mongolia | Orog Lake | 1988 | 8 | 3 | Szabóky Cs. |
| 73 | Mongolia | Orog Lake | 1988 | 8 | 3 | Szabóky Cs. |
| 74 | Mongolia | Orog Lake | 1988 | 8 | 3 | Szabóky Cs. |
| 75 | Tajikistan | Nurek | 1976 | 7 | 20 | Rácz G. |
| 76 | Tajikistan | Nurek | 1976 | 7 | 22 | Rácz G. |
| 77 | Tajikistan | Nurek | 1976 | 7 | 22 | Rácz G. |
| 78 | Tajikistan | Nurek | 1976 | 7 | 22 | Rácz G. |
| 79 | Tajikistan | Gissar Valley | 1968 | 7 |  |  |
| 80 | Uzbekistan | Charvak | 1982 | 5 | 27-6. 3 | Peregovits L. |
| 81 | Uzbekistan | Chimgan | 1988 | 6 | 13 | Vlad Štĕrba |
| 82 | Uzbekistan | Bukhara | 1934 | 4 | 18 | E. Pfeiffer |
| 83 | Uzbekistan | Bukhara | 1934 | 6 | 30 | E. Pfeiffer |
| 84 | Uzbekistan | Bukhara | 1934 | 8 | 5 | E. Pfeiffer |
| 85 | China | Altun Mountains | 1936 | 6 |  | Coll. Velez |
| 86 | Afghanistan | Kabul | 1950 | 9 |  | Coll. Velez |
| 87 | Afghanistan | Kabul | 1974 | 5 | 12 | Papp L. |
| 88 | Afghanistan | Panjao | 1968 | 8 | 5-7 | D. Müting |
| 89 | Afghanistan | Kabul | 1951 | 9 |  | Coll. Velez |
| 90 | Afghanistan | Kabul | 1951 | 9 |  | Coll. Velez |
| 91 | Turkey | Akşehir | 1928 | 6 | 1-10 | Coll. v. Bartha |
| 92 | Turkey | Akşehir | 1928 | 7 | 1-10 | Coll. v. Bartha |
| 93 | Turkey | Akşehir | 1928 | 6 | 21-30 | Coll. v. Bartha |
| 94 | Turkey | Akşehir | 1928 | 7 | 21-31 | Coll. v. Bartha |
| 95 | Turkey | Akşehir | 1928 | 7 | 1-10 | Coll. v. Bartha |
| 96 | Turkey | Akşehir | 1928 | 6 | 21-30 | Coll. v. Bartha |
| 97 | Turkey | Akşehir | 1928 | 7 | 21-31 | Coll. v. Bartha |
| 98 | Turkey | Çamlıdere | 1988 | 8 | 13 | Gyulai, Hreblay, Ronkay, Ronkay |
| 99 | Turkey | Çamlıdere | 1988 | 8 | 13 | Gyulai, Hreblay, Ronkay, Ronkay |
| 100 | Turkey | Akşehir | 1928 | 7 | 21-31 | Coll. v. Bartha |
| 101 | Turkey | Akşehir | 1928 | 7 | 1-10 | Coll. v. Bartha |
| 102 | Turkey | Çamlıdere | 1988 | 8 | 13 | Gyulai, Hreblay, Ronkay, Ronkay |
| 103 | Turkey | Tunceli | 2006 | 7 | 30-31 | Csővári Tibor |
| 104 | Turkey | Tunceli | 2006 | 7 | 30-31 | Csővári Tibor |
| 105 | Turkey | Tunceli | 2006 | 7 | 30-31 | Csővári Tibor |
| 106 | Turkey | Gaziler | 2006 | 7 | 22 | Csővári Tibor |
| 107 | Turkey | Gaziler | 2006 | 7 | 22 | Csővári Tibor |
| 108 | Turkey | Gaziler | 2006 | 7 | 22 | Csővári Tibor |
| 109 | Turkey | Çamlıdere | 1988 | 8 | 13 | Gyulai, Hreblay, Ronkay, Ronkay |
| 110 | Turkey | Çamlıdere | 1988 | 8 | 13 | Gyulai, Hreblay, Ronkay, Ronkay |
| 111 | Turkey | Çamlıdere | 1988 | 8 | 13 | Gyulai, Hreblay, Ronkay, Ronkay |
| 112 | Turkey | Çamlıdere | 1988 | 8 | 13 | Gyulai, Hreblay, Ronkay, Ronkay |
| 113 | Turkey | Çamlıdere | 1988 | 8 | 13 | Gyulai, Hreblay, Ronkay, Ronkay |
| 114 | Turkey | Çamlıdere | 1988 | 8 | 13 | Gyulai, Hreblay, Ronkay, Ronkay |
| 115 | Cyprus | Larnaka | 1935 | 8 |  | Coll. Velez |
| 116 | Cyprus | Larnaka | 1935 | 8 |  | Coll. Velez |
| 117 | Cyprus | Larnaka | 1905 | 8 | 18 | Coll. Velez |
| 118 | Cyprus | Larnaka | 1935 | 8 |  | Coll. Velez |
| 119 | Cyprus | Larnaka | 1935 | 8 |  | Coll. Velez |
| 120 | Greece | Parnassos | 1997 | 6 | 28-29 | Hácz-Juhász-Bársony |
| 121 | Greece | Paliourí | 2006 | 5 | 12 | Dányi L., Kontschán J., Murányi D. |
| 122 | Greece | Paliourí | 2006 | 5 | 12 | Dányi L., Kontschán J., Murányi D. |
| 123 | Greece | Paliourí | 2006 | 5 | 12 | Dányi L., Kontschán J., Murányi D. |
| 124 | Bulgaria | Pirin | 1976 | 6 | 30 | Podlussány L. |
| 125 | Bulgaria | Pirin | 1976 | 6 | 30 | Podlussány L. |
| 126 | Bulgaria | Pirin | 1976 | 7 | 5 | Podlussány L. |
| 127 | Bulgaria | Pirin | 1976 | 7 | 2 | Podlussány L. |
| 128 | Bulgaria | Sliven | 1929 | 6 | 4 | Pawlas |
| 129 | Bulgaria | Sliven | 1929 | 6 | 4 | Pawlas |
| 130 | Bulgaria | Sliven | 1929 | 6 | 4 | Pawlas |
| 131 | Bulgaria | Sliven | 1929 | 6 | 4 | Pawlas |
| 132 | Bulgaria | Sliven | 1929 | 6 | 4 | Pawlas |
| 133 | Bulgaria | Sliven | 1929 | 6 | 4 | Pawlas |
| 134 | Ukraine | Zhytomyr | 1920 | 5 | 19 | Prosziga |
| 135 | Russia | Khotmyzhek, Russia | 1942 | 7 | 3 | Vargha György |
| 136 | Ukraine | Zhytomyr | 1920 |  |  | Prosziga [Prozsiga!] |
| 137 | Ukraine | Zhytomyr | 1920 | 5 | 19 | Prosziga [Prozsiga!] |
| 138 | Ukraine | Zhytomyr | 1920 | 5 | 19 | Prosziga [Prozsiga!] |
| 139 | Ukraine | Zhytomyr | 1920 | 5 | 19 | Prosziga [Prozsiga!] |
| 140 | Ukraine | Raspaseyevka | 1942 | 8 | 14 | Vargha György |
| 141 | Ukraine | Raspaseyevka | 1942 | 8 | 14 | Vargha György |
| 142 | Ukraine | Raspaseyevka | 1942 | 8 | 14 | Vargha György |
| 143 | Ukraine | Raspaseyevka | 1942 | 8 | 14 | Vargha György |
| 144 | Ukraine | Raspaseyevka | 1942 | 8 | 14 | Vargha György |
| 145 | Ukraine | Raspaseyevka | 1942 | 8 | 14 | Vargha György |
| 146 | Azerbaijan | Allar | 1984 | 7 | 25 | Lukhtanov V. |
| 147 | Azerbaijan | Allar | 1984 | 7 | 25 | Lukhtanov V. |
| 148 | Azerbaijan | Allar | 1984 | 7 | 25 | Lukhtanov V. |
| 149 | Azerbaijan | Allar | 1984 | 7 | 25 | Lukhtanov V. |
| 150 | Azerbaijan | Allar | 1984 | 7 | 25 | Lukhtanov V. |
| 151 | Azerbaijan | Allar | 1984 | 7 | 25 | Lukhtanov V. |
| 152 | Russia | Kislovodsk | 1983 | 6 | 7 | Lukhtanov V. |
| 153 | Russia | Pyatigorsk | 1985 | 8 | 7 | Lukhtanov V. |
| 154 | Russia | Kislovodsk | 1988 | 8 | 8 | Lukhtanov V. |
| 155 | Russia | Pyatigorsk | 1985 | 8 | 7 | Lukhtanov V. |
| 156 | Russia | Kislovodsk | 1988 | 8 | 8 | Lukhtanov V. |
| 157 | Russia | Kislovodsk | 1988 | 8 | 8 | Lukhtanov V. |
| 158 | Iran | ‘Askarān | 2007 | 6 | 9 | Hácz T., Babits J. |
| 159 | Iran | ‘Askarān | 2007 | 6 | 9 | Hácz T., Babits J. |
| 160 | Iran | ‘Askarān | 2007 | 6 | 9 | Hácz T., Babits J. |
| 161 | Iran | ‘Askarān | 2007 | 6 | 9 | Hácz T., Babits J. |
| 162 | Iran | ‘Askarān | 2007 | 6 | 9 | Hácz T., Babits J. |
| 163 | Armenia | Yerevan | 1983 | 6 | 3 | V. Lukhtanov |
| 164 | Armenia | Geghard | 1982 | 9 | 14 | Merkl, Ronkay |
| 165 | Armenia | Yerevan | 1985 | 7 | 27 | V. Lukhtanov |
| 166 | Armenia | Jrvezh | 1982 | 9 | 13 | Merkl, Ronkay |
| 167 | Armenia | Yerevan | 1985 | 7 | 27 | V. Lukhtanov |
| 168 | Armenia | Yerevan | 1982 | 9 | 12 | Merkl, Ronkay |
| 169 | Ukraine | Hurzuf | 1962 | 9 | 1 | Podlussány L. |
| 170 | Ukraine | Hurzuf | 1962 | 9 | 2 | Podlussány L. |
| 171 | Ukraine | Crimea | 1957 | 7 | 20 | Kondakov |
| 172 | Ukraine | Crimea | 1957 | 7 | 15 | Kondakov |
| 173 | Bosnia and Herzegovina | Pazarić | 1931 | 7 | 28 | Dr Fodor |
| 174 | Bosnia and Herzegovina | Jajce | 1912 | 6 |  | v. Bartha |
| 175 | Bosnia and Herzegovina | Jajce | 1912 | 6 |  | v. Bartha |
| 176 | Bosnia and Herzegovina | Trebević | 1912 | 6 |  | v. Bartha |
| 177 | Bosnia and Herzegovina | Pazarić | 1931 | 7 | 28 | Dr Fodor |
| 178 | Bosnia and Herzegovina | Pazarić | 1931 | 7 | 28 | Dr Fodor |
| 179 | Albania | Shkodër | 1916 | 6 | 29 | v. Bartha |
| 180 | Albania | Tomorrica | 1918 | 9 | 11 | v. Bartha |
| 181 | Albania | Shkodër | 1917 | 6 | 15 | v. Bartha |
| 182 | Albania | Shkodër | 1916 | 6 | 25 | v. Bartha |
| 183 | Albania | Shkodër | 1916 | 6 | 16 | v. Bartha |
| 184 | Albania | Shkodër | 1917 | 4 | 30 | v. Bartha |
| 185 | Albania | Tomorrica | 1918 | 9 | 11 | v. Bartha |
| 186 | Albania | Shkodër | 1916 | 7 | 26 | v. Bartha |
| 187 | Albania | Shkodër | 1917 | 10 | 14 | v. Bartha |
| 188 | Albania | Shkodër | 1917 | 5 | 4 | v. Bartha |
| 189 | Albania | Korab | 1918 | 7 | 24 | Csiki |
| 190 | Albania | Korab | 1918 | 7 | 23 | Csiki |
| 191 | Italy | Trieste, Fr.V.G. | 1931 | 8 | 22 | Tasso, Schatzm, Koch |
| 192 | Italy | Trieste, Fr.V.G. | 1931 | 8 | 22 | Tasso, Schatzm, Koch |
| 193 | Italy | Trieste, Fr.V.G. | 1931 | 8 | 22 | Tasso, Schatzm, Koch |
| 194 | Italy | Trieste, Fr.V.G. | 1931 | 8 | 22 | Tasso, Schatzm, Koch |
| 195 | Italy | Duino | 1934 | 8 | 11 | Schmidt A. |
| 196 | Italy | Duino | 1937 | 7 | 2-12 | Schmidt |
| 197 | Italy | Duino | 1934 | 8 | 8-11 | Schmidt |
| 198 | Italy | Duino | 1934 | 8 | 8-11 | Schmidt |
| 199 | Italy | Duino | 1937 | 7 | 2-12 | Schmidt |
| 200 | Italy | Duino | 1937 | 7 | 3-6 | Schmidt |
| 201 | Romania | Hagieni | 1984 | 9 | 16 | Székely Levente |
| 202 | Romania | Hagieni | 1987 | 5 | 28 | Székely Levente |
| 203 | Romania | Hagieni | 1987 | 5 | 28 | Székely Levente |
| 204 | Romania | Hagieni | 1987 | 5 | 29 | Székely Levente |
| 205 | Romania | Hagieni | 1988 | 7 | 21 | Székely Levente |
| 206 | Romania | Hagieni | 1988 | 5 | 29 | Székely Levente |
| 207 | Montenegro | Zelenika | 1906 | 8 |  | Horváth |
| 208 | Croatia | Split | 1908 | 7 |  | Soós |
| 209 | Montenegro | Zelenika | 1906 | 8 |  | Horváth |
| 210 | Croatia | Rab | 1931 | 7 | 11 | Schmidt A. |
| 211 | Croatia | Kaštel Sućurac | 1929 | 7 | 12 | Schmidt |
| 212 | Croatia | Split | 1929 | 6 | 1 | Schmidt |
| 213 | Croatia | Dubrovnik | 1929 | 6 | 19 | Schmidt |
| 214 | Croatia | Dubrovnik | 1929 | 6 | 2 | Schmidt |
| 215 | Croatia | Spasovac | 1914 | 6 | 9 | Dobiasch |
| 216 | Croatia | Senj |  |  |  | Dobiasch |
| 217 | Croatia | Senj | 1914 | 4 | 12 | Dobiasch |
| 218 | Croatia | Novi Vinodolski | 1924 | 8 | 9 | Schmidt |
| 219 | Slovenia | Portorož | 1966 | 7 | 21 | Wettstein J. |
| 220 | Slovenia | Portorož | 1966 | 7 | 18 | Wettstein J. |
| 221 | Slovenia | Portorož | 1966 | 7 | 23 | Wettstein J. |
| 222 | Slovenia | Portorož | 1966 | 7 | 25 | Wettstein J. |
| 223 | Slovenia | Portorož | 1966 | 7 | 23 | Wettstein J. |
| 224 | Austria | Baden | 1932 | 8 | 10 |  |
| 225 | Austria | Baden | 1932 | 8 | 30 |  |
| 226 | Austria | Hof am Leithaberge | 1932 | 6 | 6 |  |
| 227 | Austria | Baden | 1932 | 8 | 30 |  |
| 228 | Austria | Baden | 1932 | 8 | 27 |  |
| 229 | Austria | Baden | 1932 | 7 | 28 |  |
| 230 | Austria | Baden | 1932 | 8 | 25 |  |
| 231 | Austria | Baden | 1932 | 8 | 25 |  |
| 232 | Austria | Baden | 1932 | 8 | 27 |  |
| 233 | Austria | Baden | 1932 | 5 | 29 |  |
| 234 | Austria | Baden | 1932 | 8 | 25 |  |
| 235 | Austria | Baden | 1932 | 8 | 25 |  |
| 236 | Germany | Kyffhäuser | 1963 | 7 | 30 | Vojnits A. |
| 237 | Germany | Kyffhäuser | 1963 | 7 | 30 | Vojnits A. |
| 238 | Germany | Kyffhäuser | 1963 | 7 | 30 | Vojnits A. |
| 239 | Germany | Kyffhäuser | 1963 | 7 | 30 | Vojnits A. |
| 240 | Germany | Kyffhäuser | 1963 | 7 | 30 | Vojnits A. |
| 241 | France | Herrlisheim-près-Colmar, Haut-Rhin, Alsace | 1930 | 8 | 1-15 | Ch. Fischer |
| 242 | Germany | Taunus Mountains |  |  |  | Bartha |
| 243 | Germany | Lorch |  |  |  | Bartha |
| 244 | Germany | Lorch |  |  |  | Bartha |
| 245 | Germany | Taunus Mountains |  |  |  | Bartha |
| 246 | Germany | Thuringia | 1969 | 7 | 4 | Wettstein J. |
| 247 | Germany | Thuringia | 1969 | 7 | 5 | Wettstein J. |
| 248 | France | Herrlisheim-près-Colmar, Haut-Rhin, Alsace | 1930 | 8 | 1-15 | Ch. Fischer |
| 249 | France | Herrlisheim-près-Colmar, Haut-Rhin, Alsace | 1930 | 8 | 1-15 | Ch. Fischer |
| 250 | France | Herrlisheim-près-Colmar, Haut-Rhin, Alsace | 1930 | 8 | 1-15 | Ch. Fischer |
| 251 | France | Herrlisheim-près-Colmar, Haut-Rhin, Alsace | 1930 | 8 | 1-15 | Ch. Fischer |
| 252 | France | Herrlisheim-près-Colmar, Haut-Rhin, Alsace | 1930 | 8 | 1-15 | Ch. Fischer |
| 253 | France | Herrlisheim-près-Colmar, Haut-Rhin, Alsace | 1930 | 8 | 1-15 | Ch. Fischer |
| 254 | France | Martailly-lès-Brancion | 2006 | 7 | 5 | Bálint Zs. |
| 255 | France | Burnand | 2006 | 7 | 6 | Bálint Zs. |
| 256 | France | Ozenay | 2006 | 7 | 7 | Bálint Zs. |
| 257 | France | Burnand | 2006 | 7 | 6 | Bálint Zs. |
| 258 | France | Burnand | 2006 | 7 | 6 | Bálint Zs. |
| 259 | France | Burnand | 2006 | 7 | 6 | Bálint Zs. |
| 260 | France | Burnand | 2006 | 7 | 6 | Bálint Zs. |
| 261 | France | Burnand | 2006 | 7 | 6 | Bálint Zs. |
| 262 | France | Burnand | 2006 | 7 | 6 | Bálint Zs. |
| 263 | France | Burnand | 2006 | 7 | 6 | Bálint Zs. |
| 264 | France | Burnand | 2006 | 7 | 6 | Bálint Zs. |
| 265 | France | Burnand | 2006 | 7 | 6 | Bálint Zs. |
| 266 | United Kingdom | Lewes | 1925 | 8 | 18 | Riley |
| 267 | United Kingdom | Lewes | 1925 | 8 | 18 | Riley |
| 268 | United Kingdom | Lewes | 1925 | 8 | 18 | Riley |
| 269 | United Kingdom | Lewes | 1925 | 8 | 18 | Riley |
| 270 | United Kingdom | Lewes | 1925 | 8 | 18 | Riley |
| 271 | United Kingdom | Lewes | 1925 | 8 | 18 | Riley |
| 272 | France | Levens | 1926 | 8 | 14 | Schmidt |
| 273 | France | Levens | 1926 | 8 | 14 | Schmidt |
| 274 | France | Levens | 1926 | 8 | 14 | Schmidt |
| 275 | France | Vence | 1926 | 8 | 6 | Schmidt |
| 276 | Sweden | Flisby | 1933 | 7 |  | Coll. Szabó R. |
| 277 | Norway | Saltdalen, Norway | 1881 | 7 | 11 | Schoyen |
| 278 | Finland | Viipuri | 1932 | 7 | 19 | Kannisto |
| 279 | Sweden | Flisby | 1933 | 8 |  | Coll. Szabó R. |
| 280 | Finland | Espoo | 1937 | 7 | 9 | Osmo Heikinheimo |
| 281 | Finland | Maksniemi | 1939 | 7 | 22 | Dr.Vargha |
| 282 | Spain | Villamayor | 1926 | 8 |  | Querci |
| 283 | Spain | Villamayor | 1926 | 8 |  | Querci |
| 284 | Spain | Villamayor | 1926 | 8 |  | Querci |
| 285 | Spain | Villamayor | 1926 | 8 |  | Querci |
| 286 | Spain | Villamayor | 1926 | 7 |  | Querci |
| 287 | Spain | Villamayor | 1926 | 8 |  | Querci |
| 288 | Spain | Villamayor | 1926 | 8 |  | Querci |
| 289 | Spain | Villamayor | 1926 | 8 |  | Querci |
| 290 | Spain | Villamayor | 1926 | 8 |  | Querci |
| 291 | Spain | Villamayor | 1926 | 7 |  | Querci |
| 292 | Spain | Villamayor | 1926 | 8 |  | Querci |
| 293 | Spain | Villamayor | 1926 | 8 |  | Querci |
| 294 | Italy | Tempio Pausania |  | 7 | 4 |  |
| 295 | Italy | Tempio Pausania |  | 6 | 23 |  |
| 296 | Italy | Località Foxi Murdegu | 1936 | 6 | 25 | Hartig G. |
| 297 | Italy | Località Foxi Murdegu | 1936 | 6 | 25 | Hartig G. |
| 298 | Italy | Località Foxi Murdegu | 1936 | 6 | 25 | Hartig G. |
| 299 | Italy | Catania | 1913 | 5 | 5 | v. Bartha |
| 300 | Italy | Sicily | 1913 | 4 | 27 | v. Bartha |
| 301 | Italy | Sicily | 1913 | 4 | 27 | v. Bartha |
| 302 | Italy | Castelbuono | 1913 | 4 | 29 | v. Bartha |
| 303 | Italy | Castelbuono | 1913 | 4 | 29 | v. Bartha |
| 304 | Italy | Sicily | 1913 | 4 | 27 | v. Bartha |
| 305 | France | Corte, Haute-Corse | 1925 | 7 | 16 | v. Bartha |
| 306 | France | Corte, Haute-Corse | 1925 | 7 | 16 | v. Bartha |
| 307 | France | Corsica |  |  |  | v. Bartha |
| 308 | Hungary | Szilvásvárad | 1949 | 5 | 28 | Reskovits M. |
| 309 | Hungary | Eger | 1959 | 6 | 7 | Reskovits M. |
| 310 | Hungary | Eger | 1927 | 5 | 22 | Reskovits M. |
| 311 | Hungary | Mályinka | 1957 | 6 | 16 | Reskovits M. |
| 312 | Hungary | Várvölgy | 1953 | 6 | 23 | Reskovits M. |
| 313 | Hungary | Tibolddaróc | 1956 | 6 | 2 | Gaál I. |
| 314 | Hungary | Bakonykúti | 1997 | 5 | 15 | Rácz Gábor |
